# Antifungal Liposomes Directed by Dectin-2 Offer a Promising Therapeutic Option for Pulmonary Aspergillosis

**DOI:** 10.1128/mBio.00030-21

**Published:** 2021-02-23

**Authors:** Suresh Ambati, Emma C. Ellis, Tuyetnhu Pham, Zachary A. Lewis, Xiaorong Lin, Richard B. Meagher

**Affiliations:** a Department of Genetics, University of Georgia, Athens, Georgia, USA; b Department of Microbiology, University of Georgia, Athens, Georgia, USA; Duke University Medical Center

**Keywords:** amphotericin B, antifungals, dectins, liposomes, targeted drug delivery

## Abstract

Invasive fungal diseases cause millions of deaths each year. There are currently approximately 300,000 acute cases of aspergillosis, most of which result from a pulmonary infection of immunocompromised patients by the common soil organism and opportunistic pathogen Aspergillus fumigatus. Patients are treated with antifungal drugs, such as amphotericin B (AmB). However, AmB has serious limitations due to human organ toxicity. AmB is slightly less toxic if loaded in liposomes, such as AmBisome or AmB-loaded liposomes (AmB-LLs). Even with antifungal therapy, recurrent infections are common, and 1-year fatality rates may exceed 50%. We have previously shown that coating AmB-LLs with the extracellular oligomannan-binding domain of the C-type lectin receptor Dectin-2 (DEC2-AmB-LLs) effectively targets DEC2-AmB-LLs to cell walls, exopolysaccharide matrices, and biofilms of fungal pathogens *in vitro*. *In vitro*, DEC2-AmB-LLs reduce the effective dose of AmB for 95% inhibition and killing of A. fumigatus 10-fold compared to that of untargeted AmB-LLs. Herein we tested the antifungal activity of DEC2-AmB-LLs relative to that of untargeted AmB-LLs in immunosuppressed mice with pulmonary aspergillosis. Remarkably, DEC2-AmB-LLs bound 30-fold more efficiently to A. fumigatus at sites of infection in the lungs. Furthermore, Dectin-2-targeted liposomes delivering AmB at a dose of 0.2 mg/kg of body weight significantly reduced the fungal burden in lungs compared to results with untargeted AmB-LLs at 0.2 mg/kg and micellar voriconazole at 20 mg/kg and prolonged mouse survival. By dramatically increasing the efficacy of antifungal drugs at low doses, targeted liposomes have the potential to create a new clinical paradigm to treat diverse fungal diseases.

## OBSERVATION

A. *fumigatus* is the principal causative agent of invasive aspergillosis, one of the four most common life-threatening fungal diseases. This fungus is ubiquitous, being found in soil, compost, homes, and work places. Globally, there are estimated to be approximately 300,000 acute cases of aspergillosis each year (1). In 2017, aspergillosis accounted for 17% of the U.S. costs to treat fungal infections (2). Patients at the greatest risk of developing life-threatening pulmonary aspergillosis generally have weakened immune systems and/or have various lung diseases. Three first-line clinical treatments are approved for invasive pulmonary aspergillosis: micellar voriconazole (VRZ in Vfend), isavuconazole, and liposomal amphotericin B (AmB in AmB-loaded liposomes [AmB-LLs] or AmBisome) (3). However, even with the current antifungal therapy, the fatality rates at the 1-year survival point may exceed 50%. Liposomal AmB formulations, such as AmBisome or AmB-LLs, penetrate more efficiently into various organs and show less nephrotoxicity or infusion toxicity at higher doses of AmB than do the detergent-solubilized micellar formulations (4–6). Yet, packaged in liposomes or not, AmB is used for short-term treatment due to its renal toxicity at effective doses.

**FIG 1:**
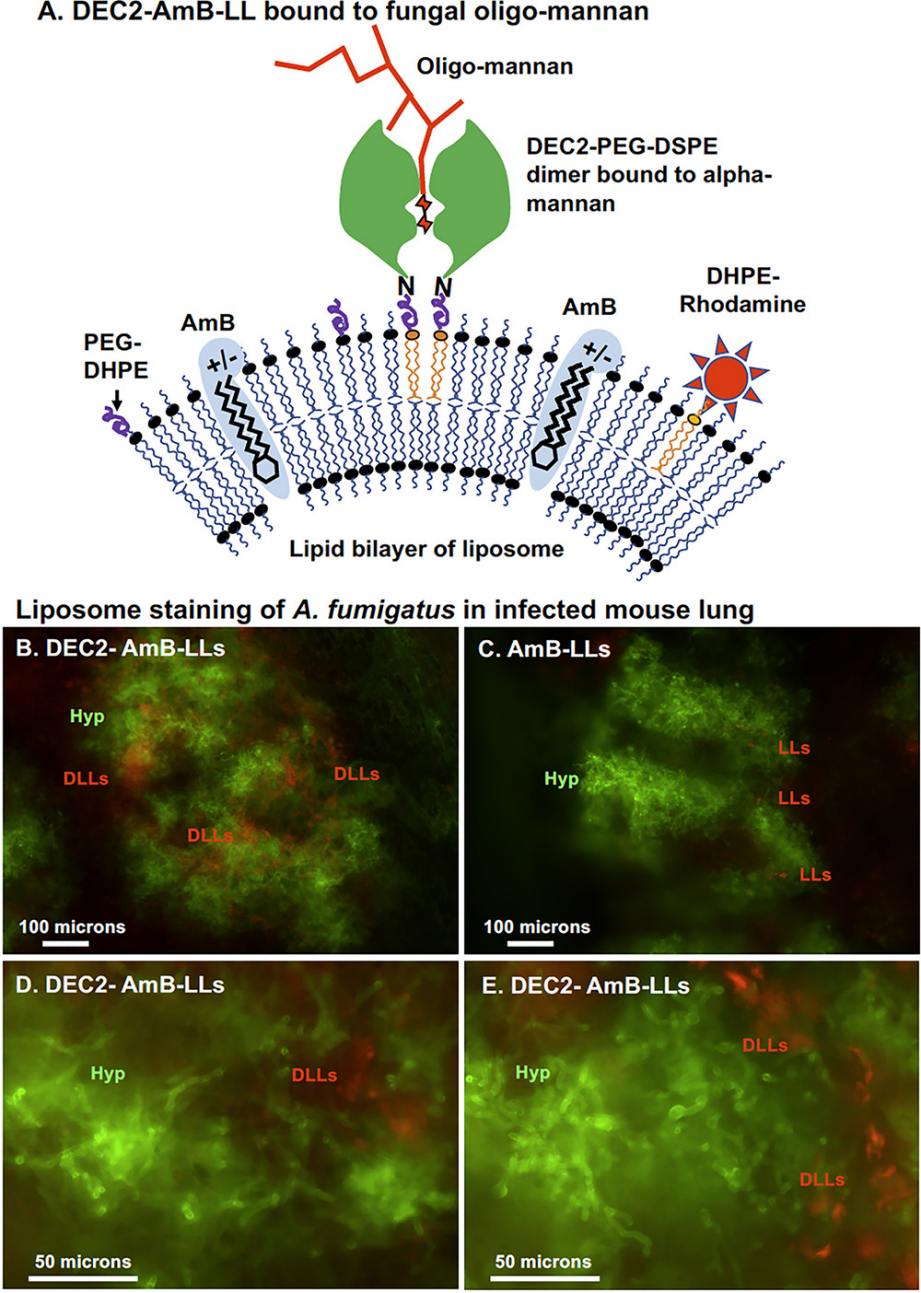
Dectin-2-coated amphotericin B-loaded liposomes (DEC2-AmB-LLs) bind A. fumigatus. (A) Model of DEC2-AmB-LL binding to the exopolysaccharide of A. fumigatus. AmB (blue ovoid structure) is amphiphilic. Its hydrophobic end is intercalated into the lipid bilayer of liposomes, as in AmBisome. Dectin-2 (green globular structure) coupled to the lipid carrier as 1,2-distearoyl-*sn*-glycero-3-phosphoethanolamine-polyethylene glycol-DEC2 (DSPE-PEG-DEC2). Two Dectin-2 monomers must float together and form dimers to bind to fungal cell wall mannans (red sugar moieties). Red fluorescent *N*-(fluorescein-5-thiocarbamoyl)-1,2-dihexadecanoyl-*sn*-glycero-3-phosphoethanolamine (DHPE)-rhodamine B (red star) was inserted into the liposomal membrane for visualization of the liposomes. This diagram is modified from those in the work of Ambati et al. (8) and Gow et al. (24). (B to E) High concentrations of DEC2-AmB-LLs (DLLs) in panels B, D, and E, but not of untargeted AmB-LLs (LLs) in panel C, were observed in fungal infection sites. Calcofluor white-stained fluorescent fungal cells were imaged in green and rhodamine B-tagged fluorescent liposomes in red. These images are representative of dozens of infection sites in which liposomes were detected in the various lungs examined. Three mice were in each treatment group, and all six lungs examined showed significant levels of infection. Replicate images and quantification are presented in Fig. S2 in the supplemental material. Hyp, hyphae.

We recently developed a potentially transformative technology that uses the carbohydrate recognition domains of C-type lectin receptors, Dectin-1 and Dectin-2, for the pan-antifungal targeting of drug-loaded liposomes to fungal beta-glucan or alpha-mannan oligosaccharides (7–9). Dectin-targeted liposomes bind specifically to the cell walls and secreted exopolysaccharide matrices of evolutionally diverse fungal pathogens, including Aspergillus fumigatus, Candida albicans, and Cryptococcus neoformans (7). Dectin-2-coated AmB-loaded liposomes (DEC2-AmB-LLs) bind to the exopolysaccharide secreted from A. fumigatus (Fig. 1). DEC2-AmB-LLs bind orders of magnitude more efficiently to A. fumigatus at all developmental stages, including conidia, germlings, and hyphae, than do untargeted liposomes, AmB-LLs (7, 8). Accordingly, DEC2-AmB-LLs inhibit and/or kill A. fumigatus 10- to 90-fold more efficiently *in vitro* (7, 8).

The goal of this study is to assess the efficacy of Dectin-2-coated liposomal AmB in mouse models of pulmonary aspergillosis. Here, we employed both steroid and neutropenic mouse models of immunosuppression (see Fig. S1 in the supplemental material). Immunosuppressed mice were infected with A. fumigatus conidia via oropharyngeal delivery and subsequently treated with DEC2-AmB-LLs, AmB-LLs, or liposome dilution buffer. The efficacy of the treatment regimen was analyzed by measuring fungal cell binding in the lungs, fungal burden in the lungs, and the rates of mouse survival.

10.1128/mBio.00030-21.1 FIG S1 Regimens of immunosuppression, infection, liposome treatments, assays of fungal burden, and assays of mouse survival. (A) Two immunosuppression mouse models of pulmonary aspergillosis, a steroid model and a neutropenic model, were used to assay the efficacy of targeted DEC2-AmB-LLs and untargeted AmB-LLs based on lung fungal burden on day 4 (D4). (B) A neutropenic model of immunosuppression was used to assay the efficacy of DEC2-AmB-LLs and AmB-LLs based on mouse survival rate. The experiment was terminated on D24. The days of treatment are indicated before and after the day of infection (D0). CP, cyclophosphamide; TC, triamcinolone. Download FIG S1, PDF file, 0.3 MB. Copyright © 2021 Ambati et al. 2021 Ambati et al. https://creativecommons.org/licenses/by/4.0/ This content is distributed under the terms of the Creative Commons Attribution 4.0 International license.

10.1128/mBio.00030-21.2 FIG S2 Antifungal-drug-loaded liposomes (DEC2-AmB-LLs), but not untargeted AmB-LLs, bound efficiently to A. fumigatus infection sites in the lung tissue. (A to D) Calcofluor white staining of fungal chitin (green) revealed infection sites in the lung that were densely packed with A. fumigatus hyphae (Hyp). Rhodamine B-tagged fluorescent liposomes are shown in red. (A and B) Untargeted AmB-LLs (LLs) were rarely localized to infection sites. (C and D) Dectin-2-targeted amphotericin B-loaded liposomes, i.e., DEC2-AmB-LLs (DLLs), were efficiently localized at infection sites. (G) The relative areas of the red fluorescent liposome signal in lung sections were quantified and compared between mice given AmB-LLs and those given DEC2-AmB-LLs (*n* = 10 images each). Download FIG S2, PDF file, 0.2 MB. Copyright © 2021 Ambati et al. 2021 Ambati et al. https://creativecommons.org/licenses/by/4.0/ This content is distributed under the terms of the Creative Commons Attribution 4.0 International license.

First, we tested for the potential preferential binding of DEC2-AmB-LLs to fungal cells in the infected lungs of neutropenic mice relative to that of AmB-LL. Mice infected with A. fumigatus on day 0 (D0) were given an oropharyngeal treatment with liposomes at day 1 postinfection (D1). The lungs were harvested at 48 h postinfection (D2). Sections taken from the dissected lungs were stained for fungal cell wall chitin and examined directly by epifluorescence microscopy. We observed multiple infection sites with extensive hyphal growth of approximately 200 to 500 μm in diameter in about half of the lung sections examined (Fig. 1B and C; Fig. S2). Significant amounts of rhodamine-labeled DEC2-AmB-LLs were observed in association with fungal cells in approximately 25% of the infection centers examined. When detected, DEC2-AmB-LLs appeared to be in patches within the infection sites and not bound directly to fungal cells themselves (Fig. 1B, D, and E; Fig. S2C and S2D), consistent with previous *in vitro* evidence that they bind primarily to the exopolysaccharide matrix (7). In contrast, AmB-LLs were barely detectable in various infection sites (Fig. 1C; Fig. S2A and S2B). In those infection sites in which liposomes were detected, the density of DEC2-AmB-LLs was 30-fold higher than the density of AmB-LLs (Fig. S2E). In summary, targeted liposomes are preferentially concentrated at sites of infection.

**FIG 2:**
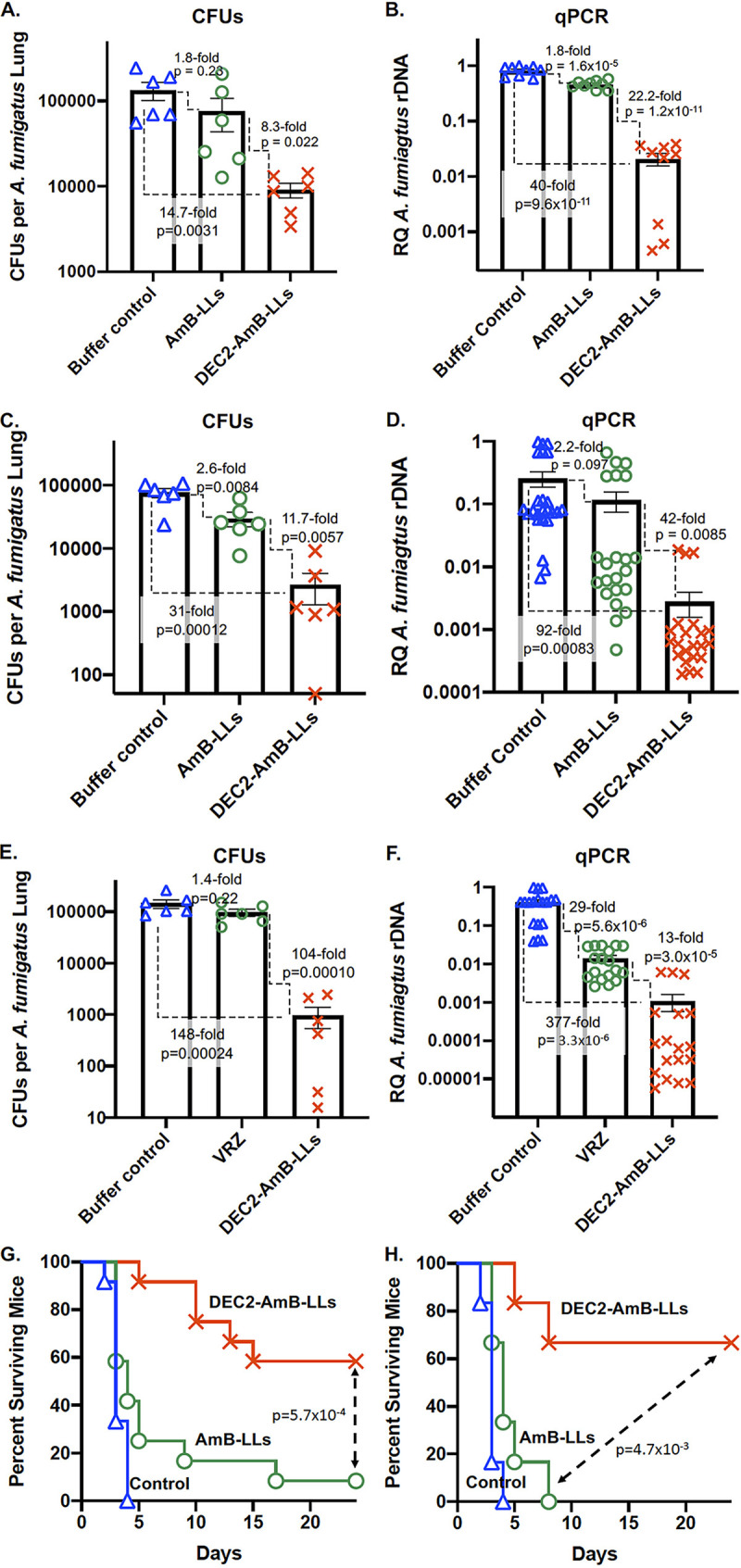
Compared to AmB-LLs, DEC2-AmB-LLs were markedly more effective at reducing fungal burden in the lungs and extending mouse survival. (A to F) Fungal burden in the lungs following liposome treatments delivering 0.2 mg/kg AmB. (A and B) Fungal burden assessment in the steroid model of immunosuppression. (A) Scatter bar plots compare the average numbers of CFUs per lung for the three treatment groups (buffer control, AmB-LLs, and DEC2-AmB-LLs), with data points showing the values for individual mice. (B) The relative quantity (RQ) of the A. fumigatus rDNA intergenic spacer was determined by qPCR of lung homogenate from the same mice. (C and D) Fungal burden assessment in the neutropenic model of immunosuppression. (C) Numbers of CFUs per lung for the three treatment groups. (D) The relative quantity of the A. fumigatus rDNA intergenic spacer was determined by qPCR of the lung homogenates from the same mice. (E and F) Fungal burdens are compared among neutropenic mice receiving two doses of 20-mg/kg VRZ and one dose of either 0.2-mg/kg AmB in DEC2-AmB-LLs or control buffer on D1 and D2. (E) Numbers of CFUs per lung. (F) Relative quantities of A. fumigatus rDNA from the same mice. (G and H) Targeted DEC2-AmB-LLs significantly improved mouse survival relative to that of mice receiving untargeted AmB-LLs. Neutropenic mice infected with A. fumigatus were treated twice with DEC2-AmB-LLs or AmB-LL delivering 0.2 mg AmB/kg or liposome dilution buffer. Mouse survival was monitored for 24 days postinfection following the regimen displayed in Fig. S1 in the supplemental material. (B) The results from two representative experiments show the percentage of mice surviving plotted against numbers of days postinfection. Twelve mice were included in each treatment group in panel E and 6 mice per treatment group in panel F. Standard errors, fold differences, and *P* values are indicated for most comparisons.

We next examined the reduction in fungal burden in the lungs after liposome treatment. Under the steroid model of immunosuppression, mice were infected with 10^6^ conidia on D0 and treated on D1 with DEC2-AmB-LLs or AmB-LLs delivering 0.2 mg AmB/kg of body weight. The liposome dilution buffer was given to the control group. On D4, the mice were euthanized and the lung tissues were homogenized. DEC2-AmB-LL-treated mice showed a significant 8.3-fold reduction in the average number of fungal CFUs in lung tissue relative to the number in AmB-LL-treated mice (*P* = 0.022) (Fig. 2). When fungal burden was assayed based on the amount of the A. fumigatus ribosomal DNA (rDNA) intergenic transcribed spacer (ITS) region using quantitative PCR (qPCR), there was a significant 22-fold reduction in fungal burden for DEC2-AmB-LL-treated mice relative to the burden in AmB-LL-treated mice (*P* = 1.2 × 10^−11^) (Fig. 2B). We noted that the steroid model was problematic in our setting, as approximately 20% of the mice died on D1 or D2 regardless of the treatment regimen from what appeared to be exacerbated lung inflammation. These mice were dropped from the analysis. Hence, the following work was performed with a neutropenic model of immunosuppression.

Infected neutropenic mice were treated on D1 with either AmB-LLs or DEC2-AmB-LLs delivering 0.2 mg AmB/kg or liposome dilution buffer (Fig. S1). On D4, the mice were assayed for fungal burden. Mice treated with DEC2-AmB-LLs showed a significant 12-fold reduction in the number of lung CFUs (*P* = 0.0057) and a 42-fold reduction in the amount of the fungal rDNA ITS (*P* = 0.0085) relative to those in AmB-LL-treated mice (Fig. 2C and D). Biological replicates of the fungal burden experiments gave similar results. In summary, in either the steroid or the neutropenic model of pulmonary aspergillosis, Dectin-2-targeted liposomal AmB significantly improved the efficacy of the drug at reducing lung fungal burden.

Given that VRZ is also used clinically as a front-line therapy against aspergillosis, we compared VRZ and DEC2-AmB-LLs in our setting. VRZ (20 mg/kg) in a micellar formulation (Vfend) delivered twice daily is as effective as AmB (5 mg/kg) in AmBisome delivered once daily in a 2-day therapy to achieve a 90% reduction in fungal burden in the lungs in a steroid mouse model of pulmonary aspergillosis (10). Here, we performed a relatively parallel experiment but used a much lower dose of AmB. We gave neutropenic mice VRZ (20 mg/kg) in a micellar formulation twice daily and AmB (0.2 mg/kg) in DEC2-AmB-LLs once daily, each for 2 days. The drop in the fungal burden of the DEC2-AmB-LL group relative to that of animals given the buffer control was estimated to be 148-fold (*P* = 0.00024) by CFU counts and 377-fold (*P* = 3.3 × 10^−6^) based on qPCR (Fig. 2E and F). As expected, treatment with two doses of DEC2-AmB-LLs was more effective at reducing fungal burden than treatment with one dose (Fig. 2C and D). Remarkably, relative to micellar VRZ at 20 mg/kg, DEC2-AmB-LLs delivering 0.2 mg/kg AmB further reduced the fungal burden 104-fold (*P* = 0.00010) based on CFUs and 13-fold (*P* = 3.0 × 10^−5^) based on qPCR (Fig. 2E and F). In short, the targeting of a low dose of liposomal AmB to *Aspergillus* significantly improved drug performance compared to that of untargeted VRZ.

To examine if reduced fungal burden correlates with prolonged host survival, we performed the animal infection and treatment study similarly to the aforementioned fungal burden studies except for the following changes: (i) mice were treated with cyclophosphamide and triamcinolone every 5 days to maintain immunosuppression (Fig. S1), (ii) mice were inoculated with 2 × 10^6^ conidia, and (iii) mice were treated with liposomes delivering 0.2 mg AmB/kg at both D1 and D2. Animal survival was monitored until D24. All 12 mice in the control group died by D4, with an average survival time of 3.25 days (Fig. 2G). AmB-LL treatment resulted in an average mouse survival time of 6.9 days, with one of the 12 mice surviving to D24. Remarkably, DEC2-AmB-LL treatment increased the average survival time to 18.4 days (*P* = 5.7 × 10^−4^), with 7 of the 12 mice showing no sign of sickness at D24 (Fig. 2E). Biological replicates of this experiment gave similar results (Fig. 2H) (*P* = 0.0047).

In a previous study, AmB-LLs coated with the monoclonal 34A targeting host pulmonary capillary cells delivering 2 mg AmB/kg, but not 1 mg/kg, reduced lung fungal burden 2-fold and improved mouse survival relative to that of mice given AmB-LLs (11). We speculate that the specific targeting of DEC2-AmB-LLs to the fungal pathogen itself rather than the host lung tissue contributed to the significantly higher efficacy that we observed here at much lower AmB doses than reported for 34A-AmB-LLs.

In brief, relative to untargeted AmB-LLs, DEC2-AmB-LLs bound more efficiently to fungal infection sites in the lungs and showed much higher efficacy in treating murine aspergillosis based on both lung fungal burden and animal survival rates. Targeted antifungal drug delivery increases efficacy at significantly lower doses of AmB. This should reduce drug toxicity and increase patient safety, leading to improved outcomes, particularly for patients infected with azole-resistant strains. It is our conviction that targeting of antifungal drugs to fungal cells will have pan-antifungal applications against diverse fungal diseases, including candidiasis, cryptococcosis, fungal keratitis, histoplasmosis, and onychomycosis (7, 12).

### Strains and growth conditions.

A. fumigatus CEA10 (13, 14) is a clinical isolate commonly employed in mouse models of pulmonary aspergillosis (15). Conidia were prepared by growing cells for 6 days at 37°C on 1.5% agar plates containing Vogel’s minimal medium (VMM) plus 1% glucose (16) plus 100 μg/ml each of kanamycin and ampicillin. Conidia were harvested in phosphate-buffered saline (PBS) plus 0.05% Tween 20, filtered through a sterile 40-μm nylon mesh filter (Fisher Scientific catalog [cat.] no. 22363547), and settled at 1 × *g* overnight to concentrate conidia. Cell density was determined in a hemocytometer, and conidia were stored at 4°C. Germination rates *in vitro* were close to 100%. Conidia were diluted to desired concentrations in PBS and vortexed just prior to being delivered to mice.

Seven- to 8-week-old outbred female CD1 (CD-1 IGS) Swiss mice were obtained from Charles River Laboratories. Mice were maintained in UGA’s Animal Care Facility. All mouse protocols met guidelines for the ethical treatment of nonhuman animals outlined by the U.S. Federal Government (17) and UGA’s Institutional Animal Care and Use Committee (AUP no. A2018 12-009).

### Immunosuppression.

In the steroid model, mice were immunosuppressed with the synthetic steroid triamcinolone acetonide (TC). In the neutropenic immunosuppression model, mice were treated with the antimetabolite cyclophosphamide (CP) in addition to TC (Fig. S1). A cyclophosphamide (Cayman cat. no. 13849) stock of 35 mg/ml was prepared in saline, pH 7.4, filter sterilized, and delivered subcutaneously at 175 mg/kg. A triamcinolone (Millipore Sigma; cat. no. T6376) stock of 40 mg/ml was prepared in dimethyl sulfoxide (DMSO) and stored at 4°C. This stock was diluted approximately 1:4 (vol/vol) in distilled water to prepare 100 μl of an aqueous suspension just prior to subcutaneous injection of 40 mg/kg. For assays of fungal burden in the steroid model, mice were given 40 mg/kg TC (18) on day −1 (D–1) (Fig. S1). Under the neutropenic model, mice were given 175 mg/kg CP on D–3 and then 40 mg/kg TC on D–1 (19). For mouse survival studies, neutropenic-model mice were also given subsequent injections using the same doses of CP and TC spaced 5 days apart (19).

### Infection.

Immunosuppressed mice were infected by oropharyngeal aspiration (20) of a 50-μl aliquot of 1 × 10^6^ to 3 × 10^6^ conidia in PBS on D0 (Fig. S1; Fig. 2). Mice that showed severe lethargy or lost 20% of their body weight were euthanized.

### Liposomes.

Nonpegylated AmBisome contains 11 mol% AmB relative to moles of lipid. Our pegylated equivalents, AmB-LLs and DEC2-AmB-LLs, each contained 11 mol% AmB and 2 mol% of the red fluorescent dye rhodamine B and were prepared as described previously (7, 8). We chose pegylated liposomes because of their greater *in vivo* stability (21). DEC2-AmB-LLs contained 1 mol% Dectin-2 tethered to the liposomal membrane by a lipid carrier. Liposomal AmB was delivered in a volume of 50 μl by oropharyngeal aspiration. Voriconazole was prepared in a micellar formulation for intravenous injection as described for Vfend (Pfizer Japan Inc., Tokyo, Japan; http://labeling.pfizer.com/ShowLabeling.aspx?id=618). As such, each mouse (∼30 g) received 0.6 mg of voriconazole (20 mg/kg; Thermo Fisher) and 9.6 mg of sulfobutyl ether beta-cyclodextrin sodium (SBECD; Millipore Sigma) dissolved in 120 μl sterile water by retro-orbital injection into the sinus venosus.

### Liposome binding to infection sites in the lungs.

Neutropenic mice infected with 3 × 10^6^ conidia were given an oropharyngeal treatment with rhodamine-tagged DEC2-AmB-LLs or AmB-LLs at 24 h postinfection. Three mice were in each treatment group. The DEC2-AmB-LL sample delivered to each mouse contained 25 μg of Dectin-2 in 50 μl, and the AmB-LL sample was diluted equivalently. Forty-eight hours postinfection, the mice were euthanized and lungs were harvested and rinsed in PBS. Hand-cut sections of approximately 1-mm thickness of the freshly harvested lungs were stained for 30 min with calcofluor white and washed twice in PBS. The 25 mM stock of calcofluor white (Blankophor BBH SV-2560; Bayer, Corp.) (5 mg in 218 μl of DMSO stored in the dark at 4°C) was diluted 1:1,000 into PBS plus 5% DMSO for fungal chitin staining. Tissue sections were pressed flat with a glass coverslip and examined under a Leica DM 6000 automated upright epifluorescence microscope. Chitin staining was photographed in the DAPI channel ex360/em470 (exposure time, 0.8 s) and falsely colored in green, while rhodamine-labeled liposome staining was photographed in the red fluorescent protein (RFP) channel ex560/em645 (exposure time, 0.4 s). Tiff images were recorded using a Hamamatsu ORCA-ER digital camera (model no. C4742-80-12AG). The area of red fluorescent liposome binding was estimated by moving the red channel from original Tiff images into ImageJ, converting them to an 8-bit black-and-white format, selecting “adjust>threshold,” and adjusting the captured area to match that in the red image area.

### Measurement of fungal burden.

Fungal burden was estimated in excised lungs on D4. Six animals were in each treatment group. Lungs were weighed and minced into pieces of approximately 1 mm^3^, mixed, and aliquoted into 25-mg samples.

**(i) CFU.** Twenty-five milligrams of lung tissue was homogenized for 60 to 90 s in 200 μl of PBS using a hand-held homogenizer (Kimble; cat. no. 749540-0000) with a blue plastic pestle (Kimble; cat. no. 749521-1500). The homogenate was spread evenly by shaking it with sterile 3-mm glass beads on YPD (yeast extract, peptone, and dextrose) agar plates containing kanamycin and ampicillin (100 μg/ml each). After a 15-h incubation at 37°C, the number of colonies (∼200 to 300 μm in diameter) were counted from the bottom of the plate on an EVOS imaging system at ×4 magnification, with 10 to 100 fields per CFU measurement. The average number of CFU for each lung was corrected for the area of the entire plate relative to each microscopic field and the weight of each lung. In the case of mice treated twice with DEC2-AmB-LLs, the CFU counts were so low that mature colonies were counted after 24 h of incubation.

**(ii) qPCR.** DNA was extracted from 25-mg parallel samples from each lung using Qiagen’s DNeasy blood and tissue kit (cat. no. 69504). As per the manufacturer’s instructions, the tissue was mixed with 180 μl of buffer ATL and 20 μl proteinase K. At this point, the protocol was modified to break fungal cell walls by adding glass beads and shaking the sample in a bead beater (Retsch MM300 laboratory mill) at the moderate speed for 10 min at room temperature. The homogenate contained some floating lipid, which was filtered out by passage through a Qiagen Shredder spin column (cat. no. 79654). At this point, we returned to the manufacturer’s protocol for DNA preparation beginning with the recommended 56°C incubation for 10 min. We typically obtained 25 μg of total DNA from 25 mg of lung tissue. qPCR was used to estimate the amount of A. fumigatus rDNA ITS sequence in 100 ng of total DNA. qPCR was performed using Bio-Rad’s CFX96 real-time system and SYBR green master mix (Applied Biosystems cat. no. 4309155). Cycle conditions included an annealing temperature of 50°C for 2 min, a melting temperature of 95°C for 2 min, and 45 cycles at 95°C for 15 s and 60°C for 1 min. The primer pair (Af18SrRNA2S forward primer, 5′-GGATCGGGCGGTGTTTCTATGA, and Af18SrRNA2A reverse primer, 5′-TTCTTTAAGTTTCAGCCTTGCGACCAT) yielded no detectable product even after 45 cycles of PCR when DNA from uninfected mouse lung tissue was examined. The relative quantity (RQ) of A. fumigatus rDNA was determined by normalizing all threshold cycle (*C_T_*) values to the lowest *C_T_* value determined for an infected control lung using the Δ*C_T_* method (22).

### Data availability.

Data were recorded and managed in Excel (v. 16.16.27). Student’s two-tailed *t* test was used to estimate *P* values (23). Line graphs and scatter bar plots were prepared in Graph Pad Prism 9 (v. 9.0.0).
