# Supplementary material for: Antifungal Liposomes Directed by Dectin-2 Offer a Promising Therapeutic Option for Pulmonary Aspergillosis
Source: mBio. 2021 Feb 23;12(1):e00030-21. doi: 10.1128/mBio.00030-21 (PMC8545082; doi:10.1128/mBio.00030-21)

Supplemental Figure 1

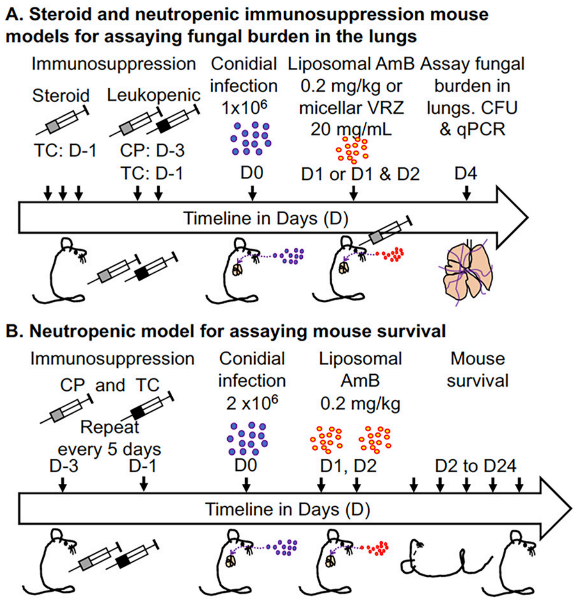

Supplement: FIG S1 [file mbio.00030-21-sf001.pdf]
